# First In-Mouse Development and Application of a Surgically Relevant Xenograft Model of Ovarian Carcinoma

**DOI:** 10.1371/journal.pone.0089527

**Published:** 2014-03-04

**Authors:** Øystein Helland, Mihaela Popa, Olav K. Vintermyr, Anders Molven, Bjørn Tore Gjertsen, Line Bjørge, Emmet McCormack

**Affiliations:** 1 Department of Obstetrics and Gynecology, Haukeland University Hospital, Bergen, Norway; 2 Department of Clinical Science, University of Bergen, Bergen, Norway; 3 Kin*N* Therapeutics, Bergen, Norway; 4 The Gade Laboratory for Pathology, Department of Clinical Medicine, University of Bergen, Bergen, Norway; 5 Department of Pathology, Haukeland University Hospital, Bergen, Norway; 6 Department of Medicine, Hematology Section, Haukeland University Hospital, Bergen, Norway; Mayo Clinic College of Medicine, United States of America

## Abstract

**Purpose:**

Preclinical models of epithelial ovarian cancer have not been exploited to evaluate the clinical standard combination therapy of surgical debulking with follow-up chemotherapy. As surgery is critical to patient survival, here we establish a combined surgical/chemotherapy xenograft model of epithelial ovarian cancer and demonstrate its translational relevance.

**Experimental Design:**

SKOV-3^luc+^ ovary cancer cells were injected topically into the ovaries of immunodeficient mice. Disease development and effect of clinical standard treatment including hysterectomy, bilateral salpingoophorectomy and removal of metastasis with follow up chemotherapy (carboplatin 12 mg/kg + paclitaxel 15 mg/kg) was evaluated by clinical parameters. Tumor burden was quantified by bioluminescence imaging (BLI).

**Results:**

The xenograft ovarian tumors developed were poorly differentiated and multicystic and the disease disseminated into the peritoneal cavity. When compared to the controls with a mean survival time of 4.9 weeks, mice treated with surgery and chemotherapy, surgery or chemotherapy demonstrated significantly improved mean survival of 16.1 weeks (p = 0.0008), 12.7 weeks (p = 0.0008), or 10.4 weeks (p = 0.008), respectively.

**Conclusion:**

Combined surgical intervention and adjuvant chemotherapy was demonstrated for the first time in an orthotopic xenograft model of ovarian cancer. Similar to observation in human studies the combined approach resulted in the longest medial survival time, advocating application of this strategy in future preclinical therapeutic development for this disease.

## Introduction

Epithelial ovarian cancer (EOC) represents about 5% of all cancers in females worldwide and is the leading cause of neoplasm-related deaths among gynecological diseases in the Western world [1]. Debulking surgery is the cornerstone in EOC treatment with the aim of maximum cytoreduction [2], [3]. First-line adjuvant chemotherapy, a combination of platinum-paclitaxel, yields response rates above 80%, including 40–60% complete responses, and improve both overall and progression free survival in all patient subgroups [4], [5]. However, most patients will later relapse and succumb to their disease due to innate or acquired drug resistance [6]. Despite improvement of surgical techniques and chemotherapeutic regimens, the overall 5-year survival rate is still below 45% [1]. New strategies, including improvement of today's standards of care, substantiated in relevant preclinical models are critical should survival rates be improved.

Preclinical evaluation of therapy in ovarian cancer has been predominantly performed in murine experimental models [7]–[9]. The syngeneic, genetically engineered mouse models (GEMM) and xenograft systems described mimic different aspects of the complexity of EOC [10]. Whereas an intact immune system in syngeneic models allows evaluation of host-tumor interactions [11], GEMM have their greatest application in unravelling the molecular basis of disease. However, a caveat in the application of GEMM relates to the relative indiscriminate nature of the genetic insertion process, which may often result in unrepresentative models of EOC. Moreover, syngeneic models are not human disease. Subsequently, results gleaned from preclinical drug screening in such systems may have questionable clinical relevance [10]. As such, xenograft EOC models of defined human cell lines are possibly a more ideal approach to study the chemo-sensitivity of both cytotoxic therapeutics and targeted agents [10], [12]. Generally, xenograft models of EOC have exploited the subcutaneous and intraperitoneal routes, owing primarily to the simplicity and ease of both inoculations of cells and to monitor therapeutic intervention. Inoculation of human cells or cell lines into orthotopic sites may nevertheless be clinically more relevant as they also replicate the early stages of tumor development [8], [10], [13], [14].

Bioluminescence imaging (BLI) has an important role in both therapeutic and molecular imaging of orthotopic xenografts of EOC [15]–[17]. However, despite advances in orthotopic xenograft model development and progression of preclinical imaging techniques of immunodeficient hosts [18], surgical intervention, i.e. the backbone of clinical therapeutic regimes in ovarian cancer [2], [19], has not been applied in a preclinical setting. Preclinical orthotopic xenografts have thus far exclusively been used to analyze the effect of cytostatics and new therapeutics [5], [20]. Therefore, the objective of this study was to evaluate surgical intervention together with a standard adjuvant chemotherapy regimen in a preclinical orthotopic xenograft model.

To achieve this we established a bioluminescent orthotopic EOC model of ovarian cancer based on the SKOV-3 cell line expressing luciferase. Xenografts disseminated into the peritoneal cavity and resulted in ascitic fluid formation analogous to what detected de novo in EOC patients. Primary tumor tissues detected by BLI were surgical removed and the effect of surgical intervention alone and/or in combination with intraperitoneal carboplatin-paclitaxel adjuvant chemotherapy in an EOC mouse model was demonstrate for the very first time.

## Materials and Methods

### Cell lines and reagents

The human ovarian adenocarcinoma cell line SKOV-3 (ATCC HTB-77) was obtained from American Type Culture Collection (ATCC, Manassas, VA, USA). The cells were cultivated in Dulbecco's modified Eagle's medium (DMEM; Gibco, Paisley, UK) supplemented with 10% heat-inactivated fetal calf serum (FCS; Gibco), 2 mM L-glutamine (Gibco) and penicillin 100 IU/ml and 100 µg/ml streptomycin (Gibco) at 37°C in a humidified atmosphere with 5% CO_2_. Cells were grown in 75 cm^2^ cell culture flasks (Costar, Cambridge, MA, USA) and subcultured twice a week. Suspensions of the cells were obtained by washing the cells twice with 10% phosphate buffered saline (PBS; Dulbecco's tablets, Oxoid Limited, Hampshire, UK) and incubating the cell cultures with Trypsin EDTA (Gibco). Thereafter, the cells were washed in growth medium, resuspended or snap-frozen for later thawing and reuse [21].

### Retroviral transfection of SKOV-3 cells

SKOV-3 clones stably expressing luciferase, denoted SKOV-3^luc+^ were engineered using the luciferase expressing construct, L192, coding for the luciferase enzyme and co-transduced with the tetracycline-regulated transactivator (tTA) which has a promoter localized upstream of L192 that drive the expression of the luciferase enzyme. Retroviral infection was performed as described earlier [22]. L192 has a puromycine resistance gene, and after two passages the cells were selected with puromycine 2 µg/ml. (Sigma-Aldrich, Oslo, Norway, stock: 25 µg/µl diluted in 0.9% sterile NaCl). Before injecting the transfected cells into animals, luciferase expression was tested. 10 µl D-luciferin (Promega, Madison, WI, USA, 150 µg/µl) was added 10 minutes prior to optical imagine to 100 µl cell suspensions containing 1×10^5^ cells in a 96-well plate (Costar).

### DNA fingerprinting

For DNA fingerprinting, genomic DNA was isolated from primary SKOV-3 cells, the SKOV-3^luc+^ cells and xenografted SKOV-3^luc+^ by the Tissue DNA kit (EZNA OMEGA Bio-tek, Norcross, GA USA, Cat.no. D3396-02) according to the manufacturer's protocol for purification of total DNA from cells. DNA concentration was determined by a Powerwave spectrophotometer by OD readings at 260 nm. The AmpFlSTR Profiler Plus PCR Amplification Kit (Applied Biosystems, Foster City, CA, USA) was employed according to the manufacturer's protocol using 1.25 ng DNA, 25 µl reaction volume and 28 amplification cycles. This kit amplifies nine tetranucleotide short tandem repeat loci and the amelogenin locus in a single reaction. Samples were run and allele sizes interpreted on an ABI 3100 Genetic Analyzer with Gene Mapper v3.7 software (both from Applied Biosystems) [23].

### Histology and immunhistochemistry

For histopathological examination, tissue sections (4 µm) were stained with hematoxylin and eosin (H&E) before they were examined by an experienced pathologist (OKM). The immunohistochemistry (IHC) staining was performed on formalin-fixed and paraffin-embedded ovarian tumor tissue sectioned at 4 µm thickness. After de-paraffination in xylene and rehydration through graded ethanol series and distilled water solution, the tissues were subjected to heat-induced epitope retrieval undertaken in TRS (Target Retrieval Solution), pH 9.9 (DakoCytomation, Copenhagen, Denmark, S3307) or citrate buffer (pH = 6.0) by the use of microwave oven at 350 W for 15 minutes. Proteinase K (Dako) endogen peroxidase activity was blocked with 0.3% peroxidase (Dako) for 5 minutes. The sections were incubated with the following primary antibodies in room temperature for 30 minutes: Monoclonal Mouse Anti-Human Cytokeratin, clone MNF116, Monoclonal Mouse Anti-Human Vimentin, clone V9, Monoclonal Mouse Anti-Human Epithelial Antigen, clone Ber-EP4, Anti-Human Wilms Tumor 1 (WT1) Protein, clone 6F-H2 (Dako, Glostrup, Denmark) and Monoclonal Mouse Anti-TAG-72, clone B72.3 (BioGenex, Fremont, USA). The staining was performed using a DAKO autostainer using the EnVision (DAKO 5007) as secondary antibody for 30 minutes for all primary antibodies. Diaminobenzine, DAB was used as chromogen for 10 minutes in development of all antibodies. Sections were counterstained with hematoxylin (Dako S3301 for 3 minutes, dehydrated and mounted in Eukitt (O. Kindler GmbH & Co, Freiburg, Germany). Negative control sections underwent the same procedure but without including primary antibody. Human tissue from high-grade serous ovarian carcinoma with known reactivity to the selected markers was used as positive control.

### Animals

The protocol for animal studies was approved by the Norwegian State Commission for Laboratory Animals (ID 3417) and the experiments were performed according to the European Convention for the Protection of Vertebrates Used for Scientific Purposes. Female NSG mice (6–8 weeks old; Vivarium, University of Bergen) were maintained under defined flora conditions in individually ventilated (HEPA-filtered air) sterile microisolator cages (Techniplast, Buguggiate, Italy) at the University of Bergen's animal facility. No more than five mice were in each individually ventilated cage that was kept on a 12 hr dark/night schedule at a constant temperature of 21°C and at 50% relative humidity. Bedding and cages were autoclaved and changed twice per month. The mice had continuous supply of sterile water and food and were monitored daily by the same personnel for the duration of the experiment and weighed three times per week. Under depilation (shaving and depilatory cream) and imaging, mice were anesthetized with 3% isoflurane (Isoba Vet, Schering-Plough, Brussel, Belgium).

### Orthotopic ovarian cancer model

Mice were anesthetized with isoflurane 3% and placed on a heating pad in lateral decubitus. The fur was clipped on the left lateral side of the abdomen, from the thoraco-lumbar junction to the iliac crest. Skin was disinfected with chlorhexidine 5 mg/ml, (Fresenius Kabi, Halden, Norway) and 70% ethanol (Kemetyl, Vestby, Norway). A 5 mm incision was made in the skin and abdominal wall, parallel and ventral to the spine, midway and between the last rib and the iliac crest. The ovarian fat pads were exteriorized and the ovaries were held in position facing the surgeon with the oviduct ventral, using a serrefine clamp. The cell suspensions (10 µL) containing 1×10^4^ SKOV-3^luc+^ cells, were inoculated inserting the needle (30 gauge) at the junction between the bursa and the fat pad. Before closing muscles and skin with continuous 5-0 monofilament non-absorbable sutures (Ethilon 5-0, Johnson & Johnson, New Brunswick, NJ, USA) the ovaries were put back to the original position. After the surgery the animals received 0.1 mg/kg buprenorphine hydrochloride (Temgesic, Reckitt Benckiser, Berkshire, UK) and were placed in a warm environment until full recovery.

### Surgery (hysterectomy, salpingoophorectomy and debulkment)

Mice were anesthetized in the same manner as for orthotopic injection and were placed in dorsal recumbence with the tail towards the surgeon. The abdominal area was shaved and swabbed with chlorhexidine and ethanol. A 2 cm midline incision was made through the skin, subcutaneous fat, muscles and linea alba. After opening the peritoneum with a scissor the abdomen was explored. Ascites, if present, was removed and staging of the cancer disease in each mouse was performed according to the FIGO system [24]. The ovaries and uterus were then removed. The ovary vein and artery were identified in mesometrium and cauterized close to the ovary on each side using a low temperature cautery, fine tip (Aaron Medical, St Petersburg, Russia). Thereafter, a single ligature with 5-0 silk suture Deknatel; (Silk-Fine Science Tools, Teleflex, NY, USA) was placed around cervix and cervix was cut with a scissor above the ligature before the uterine horns and ovaries were taken out. Any visible metastases in the peritoneum (omentum and mesentery) or adipose tissue were also eradicated. Skin and muscles were closed separately using non-absorbable suture material. The animals were kept under observation until they completely recovered from the anesthesia, and analgesics were administrated if needed [25].

### Chemotherapy

In this study, we chose an intraperitoneal route for administration of chemotherapy because it is less demanding to administrate in mice. Moreover, in human studies intraperitoneal delivery has at least the same response rate as the intravenous route and therefore are used more and more in clinical practice [26]. To determine the maximum tolerable dosage (MTD) of carboplatin (Teva, Helsingborg, Sweden 10 mg/ml) and paclitaxel (Fresenius Kabi, Halden, Norway 6 mg/ml), the following different dosages were evaluated. Carboplatin 15, 20 or 30 mg/kg, paclitaxel 12, 16 or 20 mg/kg as monotherapy or combined (*n* = 3 mice per group, total 27 mice) twice weekly for three consecutive weeks (Q2Wx3). Body weight was monitored for 28 days. A combination consisting of carboplatin 15 mg/kg together with paclitaxel 12 mg/kg was found to be the MTD. At the end of study the mice were euthanized.

### Design of trial

The mice were randomized into 4 different treatment arms with 6 mice in each group (*n* = 6): (a) control, (b) surgery alone (hysterectomy, bilateral salpingoophorectomy and removement of metastasis if present), (c) carboplatin 15 mg/kg + paclitaxel 12 mg/kg, Q2Wx3 and (d) surgery followed by carboplatin 15 mg/kg + paclitaxel 12 mg/kg Q2Wx3. Efficacy was evaluated throughout the study by BLI.

### Optical imaging (In vivo and ex vivo)

10 minutes before optical imaging with an Optix MX2 Small Animal Molecular Imager (ART Inc., Saint-Laurent, QC, Canada). Optix Optiview (version 2.00.01, ART Inc.) the mice were injected i.p. with d-luciferin (150 mg/kg). Whole body imaging in addition to imaging and examination of single organs removed after euthanasia was performed.

### Necropsy

The health status and the weight of the mice were monitored daily and mice were humanely euthanized when moribund as defined by; weight loss >10–15%, lethargy or ruffled fur. The post-mortem examination included macroscopic description of the primary tumor, metastasis and ascitic fluid. All organs were imaged ex vivo to give a further description of metastasis. The tissue biopsies were fixed in 4% buffered formalin and embedded in paraffin before they were processed for histological analysis or snap-frozen in liquid nitrogen.

### Statistical methods

Survival data was analyzed using the Kaplan and Meier method. The Mantel-Haenzel log-rank statistics (GraphPad Prism 5.0, GraphPad Software, La Jolla, CA) was used to analyze survival distribution. Survival times are quoted as mean ± standard error of the mean (SEM). Prior to initiation of the therapeutic study, xenografted mice were randomized into groups based on BLI and body weight (i.e. no individual mouse demonstrated >20% differences in either body weight or BLI from group counterparts) and ANOVA performed to ensure that there were no statistical differences between groups. For all statistical analysis, *p*<0.05 was regarded significant.

## Results

### Generation of stable, high luciferase expressing SKOV-3 cells and DNA microsatellite analysis

**Figure 1:**
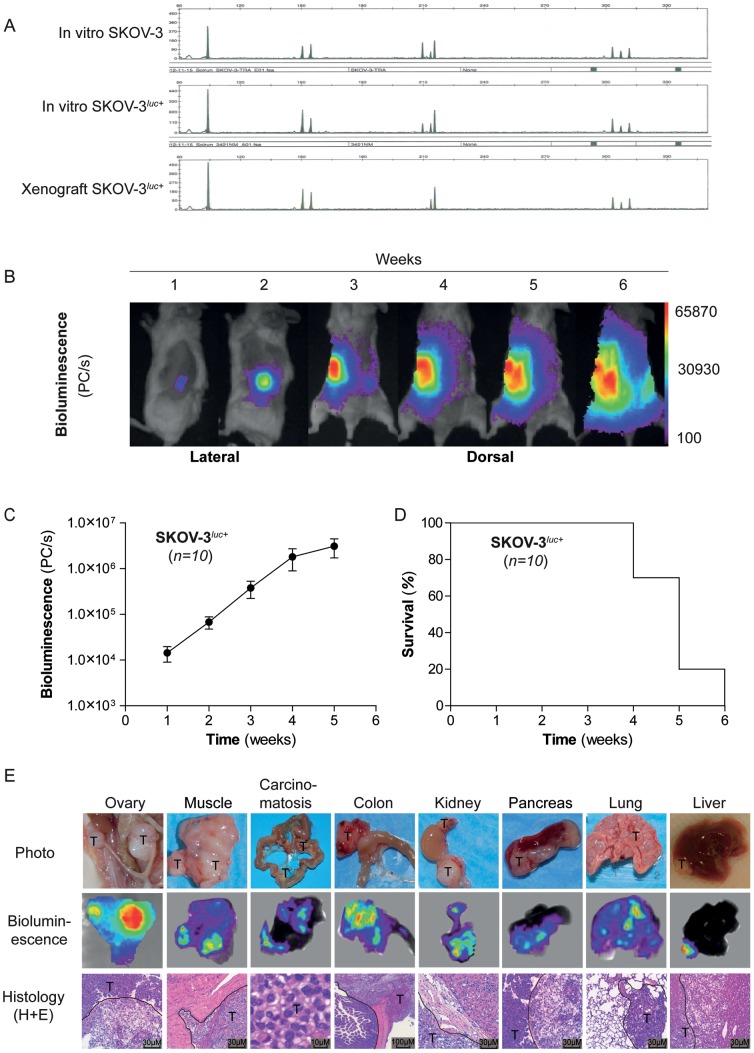
Characterization of the experimental *in vivo* mouse model. (**A**), DNA fingerprinting illustrating unique shared microsatellite DNAs between native SKOV-3, mutant SKOV-3^luc+^, and *in vivo* xenografted SKOV-3^luc+^ cells. (**B**), Illustration of *in vivo* bioluminescence imaging of orthotopic SKOV-3^luc+^ cells from one representative untreated control mouse. (**C**), Relative mean tumor growth vs time as determined by bioluminescence image analysis. (**D**), Kaplan-Meier survival curve for untreated xenografted mice (n = 10). E, Illustrations of tumor manifestations in various organs in surgical specimens (photographs), by bioluminescence imaging (BLI), and morphology (H+E). Tumors are denominated “T.” Tumor delineation against normal tissue is indicated by dashed line on H-E staining.

To develop a bioluminescent ovarian cancer cell line to permit longitudinal spatio-temporal monitoring of orthotopic xenografts, the ovarian adenocarcinoma cell line SKOV-3 was transfected with a luciferase reporter as previously described [27]. Stably transfected SKOV-3^luc+^ were selected with puromycin. The DNA microsatellite analysis of SKOV-3^luc+^ and wild-type SKOV-3 cells showed identical fingerprint patterns (Fig. 1A). Moreover, a comparison of DNA fingerprints with the data published for the SKOV-3 cell line in the ATCC database (www.atcc.org) established that the cells have the same origin.

### Orthotopic xenograft model

To generate a bioluminescent xenograft model of SKOV-3^luc+^ cells, the mice underwent a laparotomy by a 5 mm incision, the ovary was exteriorized and approximately 1×10^4^ SKOV-3^luc+^ cells were injected orthotopically into the left ovary of NSG mice. BLI was performed weekly (Fig. 1B). Initially, the bioluminescence was detected only at the injected ovary (week 1) and in the following two weeks disease progression, with infiltration and metastasis to the right ovary, was observed with a log increase in BLI signal (Fig. 1C). Full metastatic dissemination of the entire abdomen and thoracic cavity was observed from week 3 (Fig. 1B). Clinically, at the final week of follow-up, all mice had generated progressive volumes of ascites, defined by weight gain and pallor. The ascites were hemorrhagic and recovered volumes from peritoneal aspirates varied between 0.5 to 4 ml (data not shown). In general, mice orthotopically implanted with SKOV-3^luc+^ cells (*n* = 10) exhibited a consistent disease pattern with mice succumbing to terminal disease within 4.9±0.2 weeks (Fig. 1D). Previously, primary patient ovarian cancer samples have been demonstrated to exhibit genetic instability following xenograft in NSG mice, affecting reproducibility of that xenograft system [28]. DNA fingerprinting analysis of xenografted SKOV-3^luc+^ cells from our orthotopic model was consistent with the parental and luciferase transfected cell lines (Fig. 1A). At necropsy, a macro-anatomical description and ex vivo BLI analysis revealed not only primary ovarian tumors as anticipated but also extensive local and distal metastasis as described in Table 1 and illustrated by ex vivo bioluminescence imaging, photography and histology (Fig. 1E).

**Table 1:** Tumor engraftment and tumor progression.

	(%)
**Graft take**	100
**Metastatic incidence**	100
**Pelvic metastasis**	
Bladder	80
Ovary	70
**Lymph nodes metastasis**	
Para-aortic lumbar	70
Para aortic renal	50
Mesenteric	40
**Abdominal metastasis**	
Peritoneum	100
Pancreas	80
Liver	60
Kidney	80
Spleen	70
**Distal metastasis**	
Thoracic	50

Xenograft disease characteristics. Data is derived from 10 xenografted mice.

### Characterization of orthotopic tumors by histopathology and immunohistochemistry analysis

Histological analysis of the tumor grafts including the metastases showed that the mice had developed a highly invasive growing tumor with markedly pleomorphic nuclei with increased nuclear to cytoplasmic ratios.

**Figure 2:**
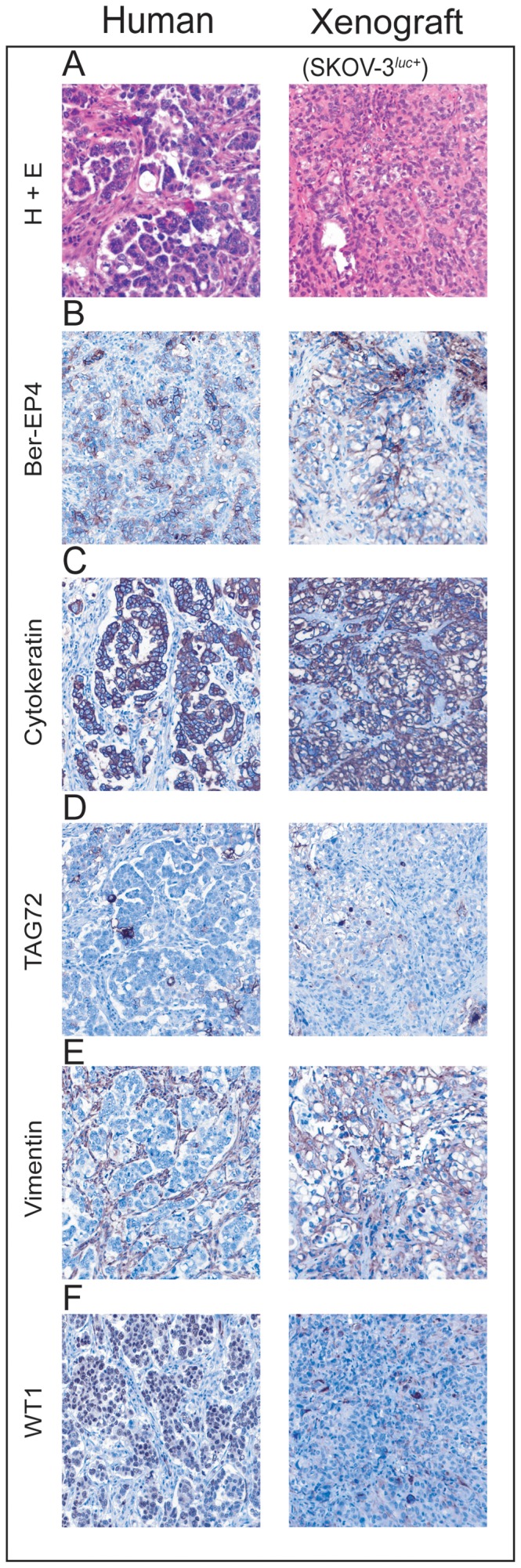
Morphological evaluation of human vs xenografted mice ovarian serous adenocarcinomas. The left column shows a high-grade serous adenocarcinoma from human ovary. The right column shows a mouse with a representative ovarian xenograft derived from human SKOV-3^luc+^ cells. (**A**), Formalin fixed paraffin embedded H+E stained sections of human (left) vs xenografted (right) mice (10× magnification), (**B–F**), Detection of various cancer protein biomarkers (Ber-EP4, cytokeratin, TAG72, vimentin, and WT1) by immunohistochemistry in human (left) vs xenografted (right) mice (10× magnification).

Immunohistochemistry analysis of both the xenografts as well as the human tumor tissue demonstrated an epithelial phenotype with positive staining for BerEp4, cytokeratin PAN and TAG-72 (Fig. 2). Only the stroma of the human tumor and not the one in the xenografts showed positive staining for Vimentin and WT-1 (Fig. 2).

### Preclinical Surgery and combination chemotherapy in a bioluminescent orthotopic xenograft model of ovarian cancer

**Figure 3:**
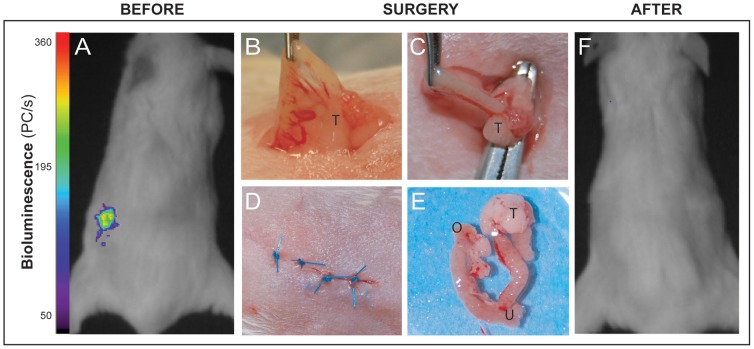
Surgical procedures and monitoring of tumor growth in xenografted mice by bioluminescence image analysis. (**A**), Preoperative bioluminescence imaging of a representative xenografted (SKOV-3^luc+^ cells) mouse (dorsal aspect) with colour bar illustrating photon counts per raster scan point (1 mm^2^). (**B–D**), Illustrations of various routine surgical procedures with exposure of right tube (B), ovary (C) and after closure of the incision in the mouse abdominal wall (D). (**E**), Routine surgical resection specimen illustrating uterus (U), ovaries (O) and the ovarian tumor (T). (**F**), Immediate postoperative (dorsal view) bioluminescent negative view indicating apparent complete surgical removal of xenografted SKOV-3^luc+^ cells.

**Figure 4:**
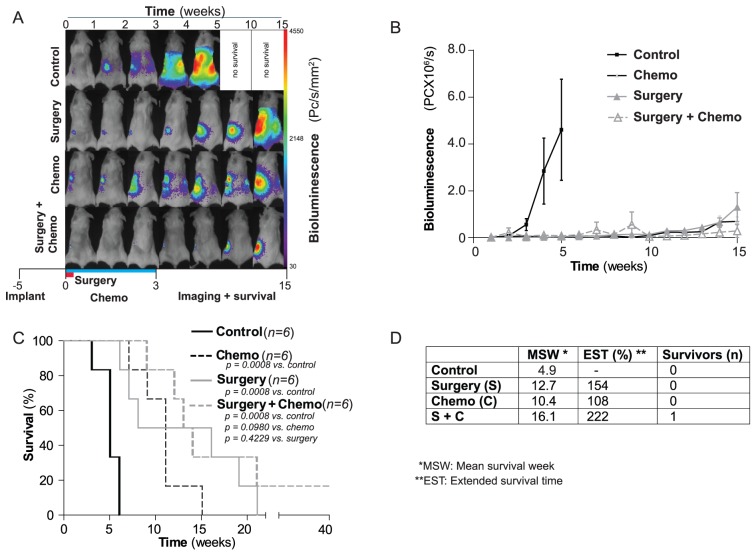
Effect of surgical treatment and chemotherapy on survival in xenografted mice. (**A**), Illustration of weekly bioluminescent image analysis of representative xenografted mice in a) control treated, b) surgical treated c) chemotherapy treated (Carboplatin and Paclitaxel) and d) surgical and chemotherapy treated mice. (**B**), Bioluminescence mean signal for the different treatment regimen with time. (**C**), Kaplan-Meyer cumulative survival curves of control, surgery, chemotherapy, a combination of surgery and chemotherapy treated mice. (**D**), Mean survival week, extended survival time (%) and number of survivors (**n**) in the variously treated groups.

The main criterion in development of this novel xenograft model of ovarian carcinoma was to enable therapeutic regimes that incorporated surgical intervention and permitted comparison of chemotherapy and surgery in the same model. Thus, 24 NSG mice were orthotopically implanted in the left ovary with 1×10^4^ SKOV-3^luc+^ cells and disease progression monitored by BLI. Following establishment of primary ovarian tumors and prior to identification of metastasis by BLI, mice were randomized into four groups each with six mice per group; (A) control, (B) combination chemotherapy with carboplatin (15 mg/kg) + paclitaxel (12 mg/kg) administered twice weekly and repeated for three weeks, (C) surgery (i.e. hysterectomy, salpingoophorectomy and evident metastasis; illustrated in Fig. 3) and (D) combined surgery and chemotherapy with therapeutic intervention monitored by BLI (Fig. 3 and 4A).

When operated, all mice had developed a tumor localized in the ovary. Most tumors were confined to the injected ovary, but three mice had already developed macroscopic visible tumors in the peritoneal lining in pelvis. According to the staging system of the International Federation of Gynecology and Obstetrics (FIGO) the mice had all stage IA to IIB.

As anticipated, all control mice developed progressive tumor growth and log increase in bioluminescence as previously observed (Fig. 4A). They were moribund within 4.9±0.5 weeks (Fig. 4 A–D). In comparison, mice treated with combination chemotherapy at week 0 and maintained on chemotherapy for three weeks, demonstrated stabilized disease for up to two weeks post-chemotherapy, before relapse to moribund condition within 10.4±1.2 weeks. In contrast, the surgical cohort initially demonstrated absence of bioluminescence in all mice following surgery on week 0. However the recurrence rate was 100% and all mice relapsed to moribund condition in 12.7±2.7 weeks. No significant differences between surgical and chemotherapeutic cohort (*p* = 0.70) was observed. Finally, the cohort treated with debulking surgery followed by adjuvant cytostatics replicated clinical response with the greatest debulkment of disease and therapeutic response. Indeed, overall survival was extended to 16.1±2.9 weeks with one mouse considered cured with no bioluminescence observed even after 40 weeks of follow-up (Fig. 4 A). While all the three treatment regimens showed significant improvement of survival rate (*p*<0.05) when compared to controls (Fig. 4 C and D), significantly curative treatment was only observed following combination of surgical intervention and cytostatics, replicating the clinical picture in EOC patients.

## Discussion

Despite being the cornerstone in the treatment of ovarian cancer, the effect of surgery has not been evaluated in preclinical models of ovarian cancer and is currently not considered when designing preclinical trials of new therapies. Here we have developed a novel bioluminescent, orthotopic xenograft, surgical model and demonstrate the application and effect of surgical debulking in combination with chemotherapy for the very first time.

Primary debulking surgery is the preferred initial treatment of women with advanced ovarian cancer [29]–[31]. Within oncology, the aggressive surgical approaches used in metastatic ovarian cancer is unique, and no other malignancies have shown demonstrable advantages of surgery in the setting of disseminated disease [19], [29]. Notably, the current preclinical EOC models have not taken this into account. Therefore, we developed an orthotopic and bioluminescent ovarian epithelial xenograft model to explore the potential of surgery in preclinical therapy development. In line with previous studies, we demonstrate that when ovarian cancer cells are injected into the unique microenvironment of the bursal membrane, a tumor xenograft was created [32]. Thereafter the tumor cells disseminated into the peritoneal cavity and a disease similar to what seen in EOC patients was established. (Fig. 1B and 1E) [8], [33]–[35]. As the use of primary patient material in xenografts has resulted in phenotypic heterogeneity and tumour cell instability we decided to use a well-defined cell line and not primary patient material [28]. The histological and immunohistochemical comparison of the xenograft and the human sample were close to identical with the exception of reduced human vimentin staining, reflecting mouse stroma, as previously demonstrated also in primary breast cancer xenografts [36], [37]. It is tempting to suggest the SKOV-3 xenograft as representative of a high-grade serous EOC (Fig. 2). However a recent study revealed the genetic profile of the SKOV-3 cells, and other frequently used cell lines, to be different from high-grade serous ovarian tumor samples. This implies that this orthotopic model has its limitations and must be evaluated in this specific context before used in preclinical studies [38]. BLI made it possible to visualise disease progression including metastatic dissemination and development of distal metastasis in liver and lungs (Fig. 1B and 4A), and the intensity of the bioluminescence signal correlated with the tumour load [27], [39]–[41]. We subsequently developed a surgical procedure (Fig. 3) permitting maximum cytoreduction, confirmed by BLI (Fig. 3F). In order to standardise the methodology and surgical procedure used, at the time of surgery all mice were operated at a lower stage of disease than most human patients at their time of clinical presentation [42]. Analogous to what has been observed clinically, surgery was the treatment modality with the greatest impact on the outcome variables (Fig. 4C and Fig. 4D) when compared to chemotherapy [43].

The group of mice treated with debulking surgery followed by adjuvant chemotherapy was, similar to observations in human studies, found to have the longest mean survival time (Fig. 4C) [44]. After maximal cytoreductive surgery, where the primary tumor is removed, there is no evidence of either local or distant metastases in patients. This was illustrated also in our model, both by the macroscopic findings after surgery and by the BLI-analysis performed after the procedure (Fig. 3F). As all mice were macroscopic tumor free after the cytoreductive surgery it was interesting to note the rather large discrepancies in disease development in the surgical cohorts with variation of ±2.7 weeks. Although we cannot rule out that sufficient cytoreduction was not achieved and also the presence of occult metastases not visible to the surgical team or BLI undetectable, micro-metastasis were the most likely cause of the early relapses. To circumvent this problem clinically, imaging (including MRI and PET/CT) has become an important facet of presurgical planning and postoperative follow-up. More recently, the emergence of fluorescence-based image-guided surgery incorporating a fluorescently labeled biomarker of an overexpressed membrane-bound protein or receptor has been successfully translated to clinical surgery of ovarian cancer [45], [46]. Preclinical development of image-guided surgery has also been performed with human cell lines inoculated subcutaneously and within the peritoneal cavity [46], [47]. While these models are not an accurate paradigm of human ovarian cancer, the application of our surgical model will now permit the realistic evaluation of image-guided surgery techniques combined with targeted drug therapy/chemotherapeutics prior to clinical translation.

Orthotopic nude mouse models have been developed for ovary carcinoma with surgical implantation of tissue [34], [48] but with less infiltrative and invasive growth compared to what is seen in our model. Although inclusion of primary patient cells isolated from ascites is a natural step in the evolution of our orthotopic model, a recent study demonstrates the complications of phenotypic heterogeneity and instability of human ovarian tumour cells [28]. Therefore we suggest that application of our reproducible cell line-based model would be more conducive in therapeutic evaluation.

In summary, we have developed a surgical orthotopic ovarian cancer xenograft model of SKOV-3^luc+^ cells resulting in a clinically relevant metastatic disease of EOC, which could be monitored by BLI. We demonstrate surgical intervention and adjuvant chemotherapy for the first time in a xenograft model of ovarian cancer, advocating this combined strategy for pre-selecting drugs regiment with greatest promise of efficacy in human clinical trials.

## Supporting Information

Figure S1 **Weight curves generated from a maximum tolerated dose (MTD) study for the combination of Carboplatin (C) and Paclitaxel (P) in NSG mice.**
(EPS)
